# miR-124 Alleviates Ischemic Stroke-Induced Neuronal Death by Targeting DAPK1 in Mice

**DOI:** 10.3389/fnins.2021.649982

**Published:** 2021-03-26

**Authors:** Yan Shi, Tian Tian, Er-Li Cai, Can Yang, Xin Yang

**Affiliations:** ^1^Faculty of Laboratory Medicine, School of Medicine, Hunan Normal University, Changsha, China; ^2^The Brain Cognition and Brain Disease Institute, Shenzhen Institutes of Advanced Technology, Chinese Academy of Sciences, Shenzhen, China; ^3^Shenzhen-Hong Kong Institute of Brain Science-Shenzhen Fundamental Research Institutions, Guangdong Key Lab of Brain Connectomics, Shenzhen, China; ^4^Britton Chance Center for Biomedical Photonics, Wuhan National Laboratory for Optoelectronics, Huazhong University of Science and Technology, Wuhan, China; ^5^Department of Emergency Surgery, Hubei Provincial Hospital of Integrated Chinese and Western Medicine, Wuhan, China

**Keywords:** stroke, cell death, miR-124, DAPK1, neuron

## Abstract

**Background:**

Ischemic stroke induces neuronal cell death and causes brain dysfunction. Preventing neuronal cell death after stroke is key to protecting the brain from stroke damage. Nevertheless, preventative measures and treatment strategies for stroke damage are scarce. Emerging evidence suggests that microRNAs (miRNAs) play critical roles in the pathogenesis of central nervous system (CNS) disorders and may serve as potential therapeutic targets.

**Methods:**

A photochemically induced thrombosis (PIT) mouse model was used as an ischemic stroke model. qRT-PCR was employed to assess changes in miRNAs in ischemic lesions of PIT-stroke mice and primary cultured neurons subjected to oxygen-glucose deprivation (OGD). 2,3,5-triphenyltetrazolium chloride (TTC) staining was performed to evaluate brain infarction tissues *in vivo*. TUNEL staining was employed to assess neuronal death *in vitro*. Neurological scores and motor coordination were investigated to evaluate stroke damage, including neurological deficits and motor function.

**Results:**

*In vivo* and *in vitro* results demonstrated that levels of miR-124 were significantly decreased following stroke, whereas changes in death-associated protein kinase 1 (DAPK1) levels exhibited the converse pattern. DAPK1 was identified as a direct target of miR-124. N-methyl-D-aspartate (NMDA) and OGD-induced neuronal death was rescued by miR-124 overexpression. Upregulation of miR-124 levels significantly improved PIT-stroke damage, including the overall neurological function in mice.

**Conclusion:**

We demonstrate the involvement of the miR-124/DAPK1 pathway in ischemic neuronal death. Our results highlight the therapeutic potential of targeting this pathway for ischemic stroke.

## Introduction

Stroke is an acute cerebrovascular disease caused by insufficient blood flow to the brain. Stroke can result in neurological dysfunction and several neurodegenerative diseases (Hu et al., 2017; Poredos et al., 2020). Strokes are either hemorrhagic or ischemic. Ischemic stroke accounts for the majority of all strokes and is the second leading cause of death worldwide (Feigin et al., 2016; Rosing et al., 2020). Transient cerebral ischemia differs from transient ischemic damage in other tissues, and the former can lead to neuronal death (Xu et al., 2016). Notably, neuronal death after ischemia is the main cause of disability and death in stroke patients (Gonzalez-Nieto et al., 2020). In ischemic stroke, cells in the core of the stroke die within the first few hours, and cells in the surrounding penumbra experience reversible damage, which persists for several hours to days, ultimately resulting in cell death (Nakka et al., 2008). The complex cell death pathways underscoring ischemic neuronal death are not fully understood. Clarifying the specific pathways involved in ischemic neuronal death may facilitate the development of novel therapeutic strategies for ischemic stroke.

MicroRNAs (miRNAs) are non-coding RNAs with lengths of 18 to 22 nucleotides. miRNAs participate in the posttranscriptional regulation of gene expression and are involved in neurogenesis, development, and maintenance of neuronal phenotype (Kaur et al., 2013; Yapijakis, 2020). miRNA-dependent regulation of neuronal survival and ischemic stroke is a research topic of growing interest (Guo et al., 2017; Lv et al., 2019; Wu F. et al., 2019). Several studies have reported that a considerable number of miRNAs are altered following focal cerebral ischemia, some of which have been implicated in the modulation of inflammation (Jaenisch et al., 2016), autophagy (Zhao et al., 2017), apoptosis (Zhang and Meng, 2019), and oxidative stress (Kanagaraj et al., 2014) by regulating target protein levels. Hence, miRNAs are considered promising therapeutic targets and have the potential to be used as diagnostic and prognostic biomarkers in cerebral ischemic stroke (Khoshnam et al., 2017). However, the specific miRNAs involved in the pathological events of neuronal death and cerebral ischemic damage have not been elucidated. miR-124 is one of the most abundant miRNAs in the central nervous system (CNS) and has been reported to regulate apoptosis and autophagy (Sun et al., 2013; Liu K. et al., 2017). miR-124 levels were significantly altered following cerebral ischemic damage (Weng et al., 2011), and abnormal miR-124 concentrations were detected in the plasma of patients with ischemic stroke (Liu X. et al., 2019). These data indicate that miR-124 may play a critical role in ischemic stroke damage.

Death-associated protein kinase 1 (DAPK1) was originally identified to be involved in cell apoptosis induced by interferon-γ. Subsequent studies reported its involvement in several cell death-related signaling pathways (Bialik and Kimchi, 2006; Kim et al., 2019). The substrates and molecular signaling pathways associated with DAPK1 in stroke damage are gradually being canvassed, but the mechanisms underpinning changes in DAPK1 levels remain unclear (Wang et al., 2020).

In this study, we identified a novel miR-124/DAPK1 pathway in neuronal death and damage following ischemic stroke. Both *in vivo* and *in vitro* results revealed that levels of miR-124 were significantly decreased following stroke. miR-124 directly targeted the 3′-UTR of DAPK1. Furthermore, down-regulation of miR-124 induced upregulation of DAPK1 protein levels, thereby inducing neuronal death and stroke damage. We further confirmed that the upregulation of miR-124 levels rescued N-methyl-D-aspartate (NMDA)/oxygen-glucose deprivation (OGD)-induced neuronal death *in vitro*. To rescue these effects, we administered an agonist, agomiR-124, to upregulate the decreased miR-124 levels in photochemically induced thrombosis (PIT)-stroke mice. Our results highlight a novel therapeutic target for ischemic stroke.

## Materials and Methods

### Animals

Male C57BL/6J mice (90 ± 5 days, 25–28 g) were used for PIT operation. All animal care and experiments were conducted in accordance with the National Institutes of Health and were approved by the Ethics Committee of Hunan Normal University.

### Cerebral Ischemia Mouse Model

Photochemically induced thrombosis-type cerebral ischemia, also known as photothrombosis, was performed as previously described (Bidmon et al., 2001). A photothrombosis model with fine control was generated using 532 nm light via an optical probe positioned above the motor cortex, which differs from traditional large cold light placed above the skull. Mice were anesthetized with 2% isoflurane and mechanically ventilated with oxygen-balanced vaporized isoflurane. Lidocaine (0.1 ml, 0.2%) was injected under the scalp, and mice also received a 0.5 ml subcutaneous injection (SC) of a saline solution containing buprenorphine (2 mg/ml), atropine (3 μg/ml), and glucose (20 mM). After the skull contralateral to the dominant side forelimb was exposed, a fiber optic bundle (200 μm diameter, 10 mW, 532 nm) was positioned within the motor cortex area (0.5 mm anterior to bregma and 0.6 mm lateral to the midline). The animal received a second SC injection of saline (0.5 ml) with 20 Mm of glucose and was allowed to recover in the home cage with an overhead heat lamp. Rose Bengal dye (10 mg/kg; Wako, Osaka, Japan, Cat# 184-00272) was injected intraperitoneally (Joa et al., 2020; Sasaki et al., 2020), and 532 nm light was delivered into the brain via the optic probe for 20 min. Sham mice received the same dose of Rose Bengal dye and underwent the same surgery but without irradiation. All mice survived the procedure.

### Neurological Status Assessment

Neurological status was assessed using modified 7-point neurological scales following PIT-stroke (Tu et al., 2010). Neurological deficit scores were: 0 = no observable neurological deficits; 1 = flexion of the contralateral torso; 2 = circling to the ipsilateral side; 3 = circling to the ipsilateral side; 4 = rolling to the ipsilateral side; 5 = leaning to the ipsilateral side at rest; 6 = longitudinal spinning; and 7 = no spontaneous motor activity/death.

### Rotarod Test

Motor coordination of mice before and after the operation and treatment was assessed using a rotarod treadmill (Ugo Basile). Mice were placed on an accelerating rotor (10 speeds from 4 to 40 rpm for 5 min). A trial ended if the mice fell off the rungs, and this was recorded as the retention time (Pei et al., 2015). Mice were trained for 2 days (three trials per day), and the mean duration was recorded. Tests were performed at 1, 3, and 7 days after PIT.

### 2,3,5-Triphenyltetrazolium Chloride Staining

At indicated time points after PIT, mice were euthanized and their brains were removed rapidly and frozen at −20°C for 15 min. Coronal slices were collected on ice. Sections were incubated in 1% TTC and protected from light at 37°C for 30 min. The presence of infarctions was determined by assessing areas devoid of TTC staining.

### Primary Neuronal Culture and Oxygen-Glucose Deprivation

Primary cultured neurons were prepared from 17- or 18-day-old mouse embryos. The cortex of embryos was dissected in Hank’s buffered saline solution and digested with EDTA-free trypsin at 37°C for 15 min. Neurons were dissociated with DMEM/F12 medium containing 10% (vol/vol) fetal bovine serum (FBS) and plated onto poly(D-lysine)-coated (Sigma, St. Louis, MO, United States) coverslips in a 12-well plate or tissue culture dish. Cells were maintained in a humidified incubator (37°C, 5% CO_2_) for 2 h, and the medium was changed to a maintenance medium [Neurobasal containing 2% (vol/vol) B27 and 1% (vol/vol) GlutaMAX]. Half of the medium was replaced with a fresh maintenance medium [Neurobasal containing 2% (vol/vol) B27] every 3 days.

After 14 days *in vitro* (DIV), primary cultured neurons were challenged with OGD. The medium was first treated with deoxygenated glucose-free Hanks’ balanced salt solution (Invitrogen). The culture plates were then transferred to a hypoxic chamber (37°C, 5% CO_2_, and 95% N_2_) for 1 h. The medium was subsequently changed to a glucose-containing medium containing 10% (vol/vol) FBS and maintained under normoxic conditions for 24 h (37°C, 5% CO_2_).

### Western Blotting

After washing with cold phosphate-buffered saline (PBS), tissues and cells were homogenized with a mixture of pH 7.4 RIPA buffer (1% Triton X-100, 10 mM Tris, 150 mM NaCl, 1 mM EGTA, 1 mM EDTA) and protease inhibitor, and centrifuged at 8,000 rpm for 5 min at 4°C. A bicinchoninic acid (BCA) kit (Thermo Fisher Scientific, Waltham, MA, United States) was used to measure the extracted proteins. Proteins were electrophoresed using 10% sodium dodecyl sulfate polyacrylamide gel electrophoresis (SDS-PAGE) and transferred onto nitrocellulose (NC) membranes. NC membranes were blocked with 5% milk dissolved in Tris-buffered saline (TBS) for 30 min. They were then incubated with primary antibodies against DAPK1 (Cell Signaling Technology, 1:1,000), DM1A (Abcam, 1:2,000), caspase-3 (Cell Signaling Technology, 1:1,000), cleaved caspase-3 (Cell Signaling Technology, 1:500), p35/25 (Cell Signaling Technology, 1:1,000), ERK1/2 (Cell Signaling Technology, 1:1,000), p-ERK1/2 (Cell Signaling Technology, 1:1,000), CaMK2 (Thermo Fisher Scientific, 1:1,000), and p-CaMK2 (Thermo Fisher Scientific, 1:1,000) at 4°C overnight. This was then followed by incubation with anti-mouse or anti-rabbit IgG conjugated to IRDye^®^ 800 CW (Li-Cor Bioscience, Lincoln, NE, United States, 1:10,000) at 37°C for 1 h. NC membranes were visualized using the Odyssey Infrared Imaging System (Li-Cor Bioscience, United States).

### RNA Extraction and qRT-PCR Analysis

Total mRNA and miRNAs were extracted with TRIzol reagent (Invitrogen, Carlsbad, CA, United States) or miRNeasy Mini Kit (Qiagen, Hilden, Germany), respectively. For mRNA and miRNA analysis, GAPDH and U6 were used as endogenous controls for normalization, respectively. The primer sequences used were as follows:

miR-124 forward, 5′-GGCATTCACCGCGTGCCTTA-3′;

miR-124 reverse, 5′-GCTGTCAACGATACGCTACG-3′;

U6 forward, 5′- GATGACACGCAAATTCGTGAA-3′;

U6 reverse, 5′- GCTGTCAACGATACGCTACG-3′;

DAPK1 forward, 5′- TTCTGTTGCTATGACTACTTTGC TG-3′;

DAPK1 reverse, 5′- AGGATGTATCCTTGTCATATCCA AA-3′;

GAPDH forward, 5′-GTCAACGGATTTGGTCTGTATT-3′;

GAPDH reverse, 5′-AGTCTTCTGGGTGGCAGTGAT-3′.

### Luciferase Activity Assay

Human embryonic kidney 293 cells (HEK293) were cotransfected with a control vector or miR-124, wild-type (WT), or mutant DAPK1 3′-UTR plasmids (psiCHECK-2 vector). After 48 h, cells were harvested and centrifuged at 12,000 rpm for 1 min, and the supernatant was collected. Luciferase activity was detected using the dual luciferase reporter assay system (E1910, Promega, Madison, WI, United States). For each cell sample, 100 μL of firefly luciferase working solution was added to detect firefly fluorescence signal, and 100 μL of Renilla luciferase working solution was added to detect the Renilla fluorescence signal. Normalized values (Renilla/firefly activity) were used for statistical analysis among groups.

### Cell Counting Kit-8 Assay

Neuronal suspensions were added to a 96-well plate at a suitable density and maintained in a humidified incubator (37°C, 5% CO_2_). After 14 DIV, cells were divided into different groups and exposed to the corresponding experimental conditions. Cells were subsequently incubated in a medium containing cell counting kit-8 (CCK8) solution (Dojindo Laboratories, Kumamoto, Japan). After incubation for 1 h, a BioTek Synergy 2 microplate reader (Winooski, VT, United States) was used to measure optical densities.

### Immunofluorescence Staining

After washing in PBS for 5 min, cultured neurons were fixed in 4% paraformaldehyde for 30 min. Cells were then washed in PBS supplemented with 0.5% Triton for 15 min, incubated in 3% BSA for 30 min, and washed in PBS for 15 min. Cells were incubated overnight at 4°C with primary antibody (anti-MAP2, Sigma, 1:1,000) for 24 h followed by incubation with secondary antibody AlexaFluor 488-conjugated goat anti-rabbit IgG (1:500, Jackson, United States) for 1 h at RT. Cells were incubated with 4′,6-diamidino-2-phenylindole (DAPI, Life Technologies) for 5 min. Fluorescence images were observed using a microscope (Olympus, Tokyo, Japan).

### Statistical Analysis

All data are expressed as mean ± standard deviation (SD) and were analyzed using SPSS version 20.0 (SPSS Inc., Chicago, IL, United States). An unpaired *t*-test was applied for comparison between two groups. Comparison of more than two groups was performed using one-way analysis of variance (ANOVA) followed by Tukey’s multiple comparisons test. Differences were considered significant if *p* < 0.05.

## Results

### PIT-Stroke Induced Decreased Levels of miR-124 and Increased DAPK1 Levels

**FIGURE 1:**
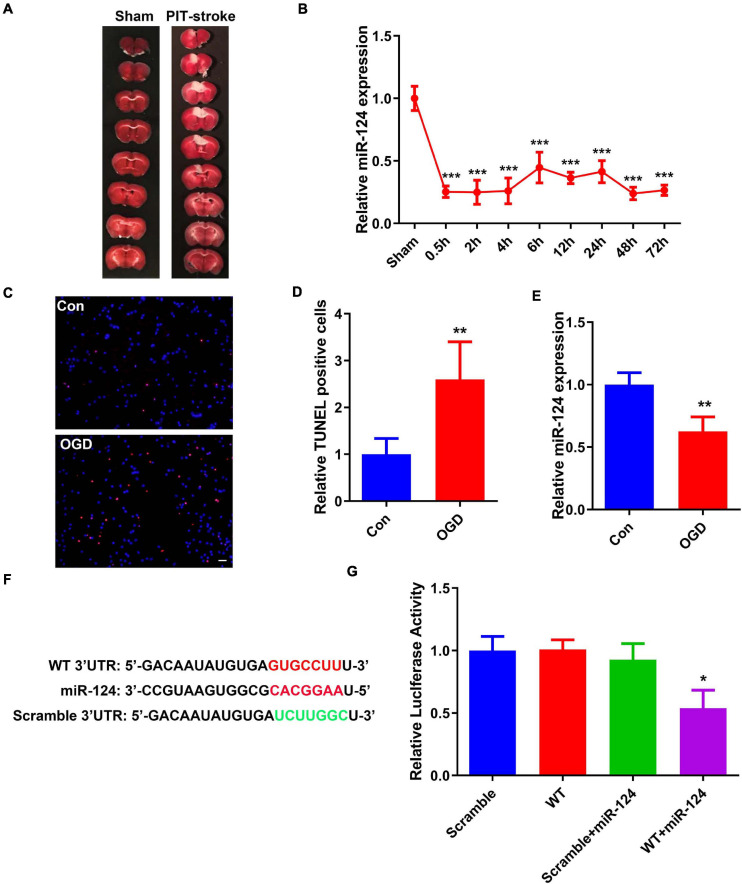
PIT-stroke induced decreased levels of miR-124 and increased DAPK1 levels. **(A)** Representative graph of 2,3,5-triphenyltetrazolium chloride (TTC) staining showing cerebral infarct in PIT-stroke. **(B)** Changes in miR-124 expression levels at 0.5, 2, 4, 6, 12, 24, 48, and 72 h after PIT-stroke (*n* = 3/group). **(C)** TdT-mediated dUTP nick end labeling (TUNEL) staining (red) in primary cultured neurons [14 days *in vitro* (DIV)] was performed after an oxygen glucose deprivation (OGD) challenge (scale bar = 50 μm) and quantified **(D)** (*n* = 4/group). **(E)** Bar graph shows the relative expression of miR-124 following OGD. **(F)** Predicted binding sites of miR-124 in DAPK1 3′-UTR and its mutant. **(G)** Human embryonic kidney 293 (HEK293) cells were co-transfected with miR-124 or control miRNA with wild-type (WT) or mutated (scrambled) 3′-UTR of DAPK1. Data are presented as mean ± SD (*n* = 6/group). *p < 0.05, **p < 0.01, ***p < 0.001 vs. Sham or Con or scramble group.

miR-124 is one of the most abundant miRNAs in the CNS and abnormal levels may result in neurological dysfunction and neurodegeneration (Liu X. et al., 2019). In this study, the PIT-stroke model was verified by TTC staining (Figure 1A and Supplementary Figure 1). To elucidate the role of miR-124 signaling in stroke damage, qRT-PCR was employed to analyze miR-124 levels in brain infarction tissues. miR-124 levels were significantly decreased at different post-stroke time points (e.g., 0.5, 2, 4, 6, 12, 24, 48, and 72 h) compared to those in sham tissues (Figure 1B).

To further examine stroke-related changes in miR-124, an *in vitro* cellular model of ischemic stroke in neurons was established, termed the neuronal OGD model. Primary cultured neurons (DIV 14) were challenged with OGD (Supplementary Figure 2). The number of TUNEL-positive neurons was significantly increased (Figures 1C,D). Consistent with the changes following PIT-stroke in mice, miR-124 was downregulated 24 h after OGD (Figure 1E).

In order to further identify the detailed mechanisms underlying miR-124-induced stroke damage, several miRNA databases were probed to predict miR124-mRNA interactions. We identified a 7-bp binding site of miR-124 located in the 3′-UTR of DAPK1 (other mRNA data not shown) (Figure 1F). Next, we verified whether miR-124 binds to DAPK1 using a luciferase reporter assay. Luciferase reporter activity was significantly decreased in the group that contained miR-124 and DAPK1 3′-UTR downstream, whereas no changes were observed in the other three groups (Figure 1G).

### DAPK1 Was Identified as a Target Gene of miR-124

**FIGURE 2:**
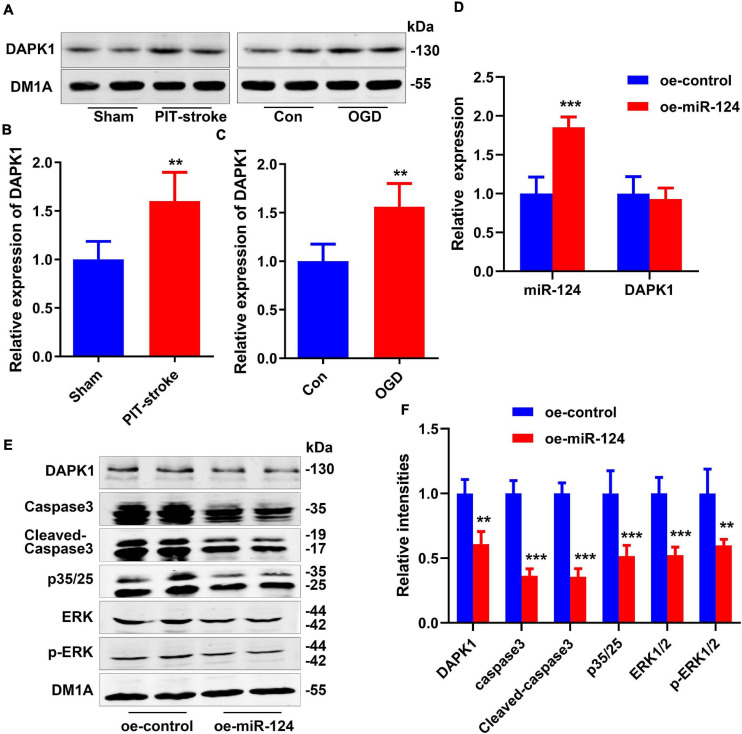
DAPK1 was identified as a target gene of miR-124. DAPK1 levels were measured by western blotting **(A)** and quantitatively analyzed **(B,C)**. Data are presented as mean ± SD (*n* = 4/group). **p < 0.01, ***p < 0.001 vs. Con or Sham group. N2a cells were transfected with miR-124 (oe-miR-124) or control-treated (oe-control) cells. Levels of miR-124 and DAPK1 mRNA were measured by qRT-PCR **(D)**. Levels of DAPK1, caspase-3, cleaved caspase-3, p35/25, ERK1/2, and p-ERK1/2 were measured by western blotting **(E)** and quantitatively analyzed **(F)**. Data are presented as mean ± SD (*n* = 4/group). **p < 0.01, ***p < 0.001 vs. oe-control group.

We next assessed whether levels of DAPK1, a downstream gene of miR-124, were altered post-stroke. Western blot revealed that levels of DAPK1 protein were significantly higher in PIT-stroke mice than in sham mice (Figures 2A,B) and significantly higher in OGD neurons than in control neurons (Figures 2A,C).

Overexpression of miR-124 in N2a cells significantly reduced DAPK1 protein levels but not mRNA levels (Figures 2D–F). Protein levels of caspase-3, cleaved Caspase-3, p35/25, extracellular regulated protein kinases 1/2 (ERK1/2), and p-ERK1/2 were significantly decreased in the miR-124 group compared to the control group (Figures 2E,F). The high expression of these proteins was closely related to cell death.

### miR-124 Exerted Protective Effects on Neuroexcitatory Toxicity

**FIGURE 3:**
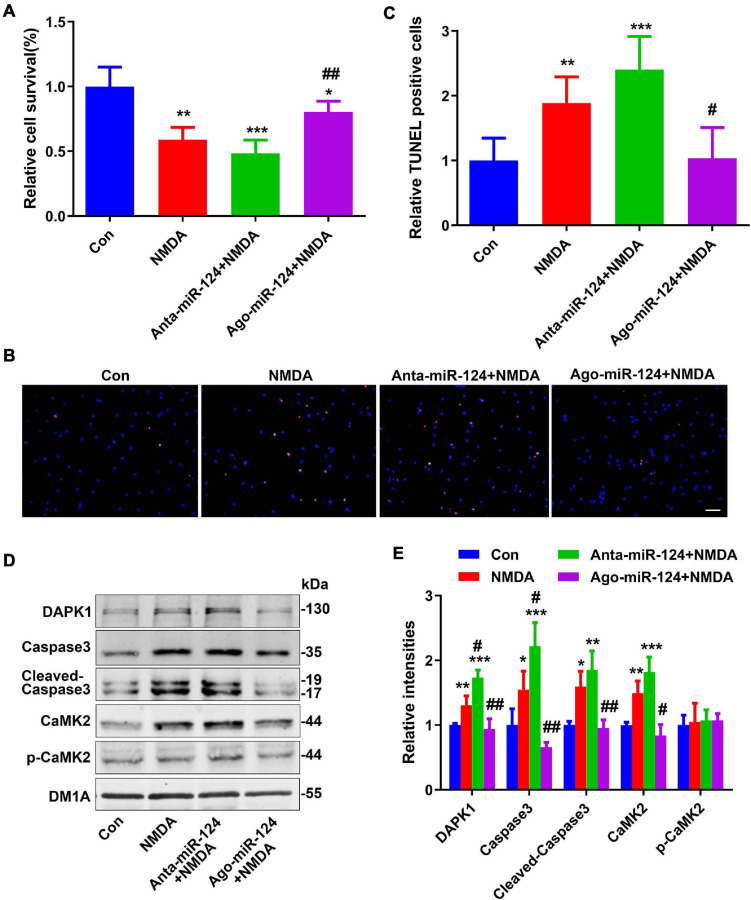
Protective effect of miR-124 on neuroexcitatory toxicity. **(A)** The relative cell viabilities of primary cultured neurons were evaluated using a cell counting kit-8 (CCK8) assay after NMDA treatment (20 μM for 15 min) (*n* = 6/group). **(B)** TUNEL staining was performed in primary cultured neurons after NMDA treatment with different miR-124 levels (scale bar = 50 μm) and quantified **(C)**. Levels of DAPK1, caspase-3, cleaved caspase-3, CaMK2, and p-CaMK2 were measured by western blotting **(D)** and quantitatively analyzed **(E)**. Data are presented as mean ± SD (*n* = 4/group). *p < 0.05, **p < 0.01, ***p < 0.001 vs. Con, ^#^*p* < 0.05, ^##^*p* < 0.01 vs. NMDA group.

Excitotoxicity mediated by NMDA receptors (NMDARs) has been proposed as a major mechanism underscoring neuronal damage after stroke (Kalotra and Kaur, 2020). Here, *in vitro* excitotoxic cell death experiments were conducted to evaluate the neuroprotective effects of miR-124 (Tran, 2019; Yamada et al., 2019). Following NMDA treatment with different miR-124 levels, the relative cell viabilities of neurons were evaluated using a CCK8 assay and TUNEL staining. Relative cell viabilities were significantly decreased (Figure 3A), whereas the number of TUNEL-positive neurons was significantly increased (Figures 3B,C) in NMDA-treated neurons. Compared to NMDA-treated neurons, antagomiR-124 (Anta-miR-124) + NMDA-treated neurons exhibited lower cell viability (Figure 3A) and greater TUNEL-positive staining (Figures 3B,C). Compared to NMDA-treated neurons, agomiR-124 (Ago-miR-124) + NMDA-treated neurons exhibited higher cell viability (Figure 3A) and less TUNEL-positive staining (Figures 3B,C).

Protein levels of DAPK1, caspase-3, cleaved caspase-3, and CaMK2 were significantly increased in NMDA-treated neurons (Figures 3D,E). Compared to levels in NMDA-treated neurons, the levels of these proteins were significantly increased in Anta-miR-124 + NMDA-treated neurons and significantly decreased to normal levels in Ago-miR-124 + NMDA-treated neurons (Figures 3D,E).

### miR-124 Protected Against PIT-Stroke Damage

**FIGURE 4:**
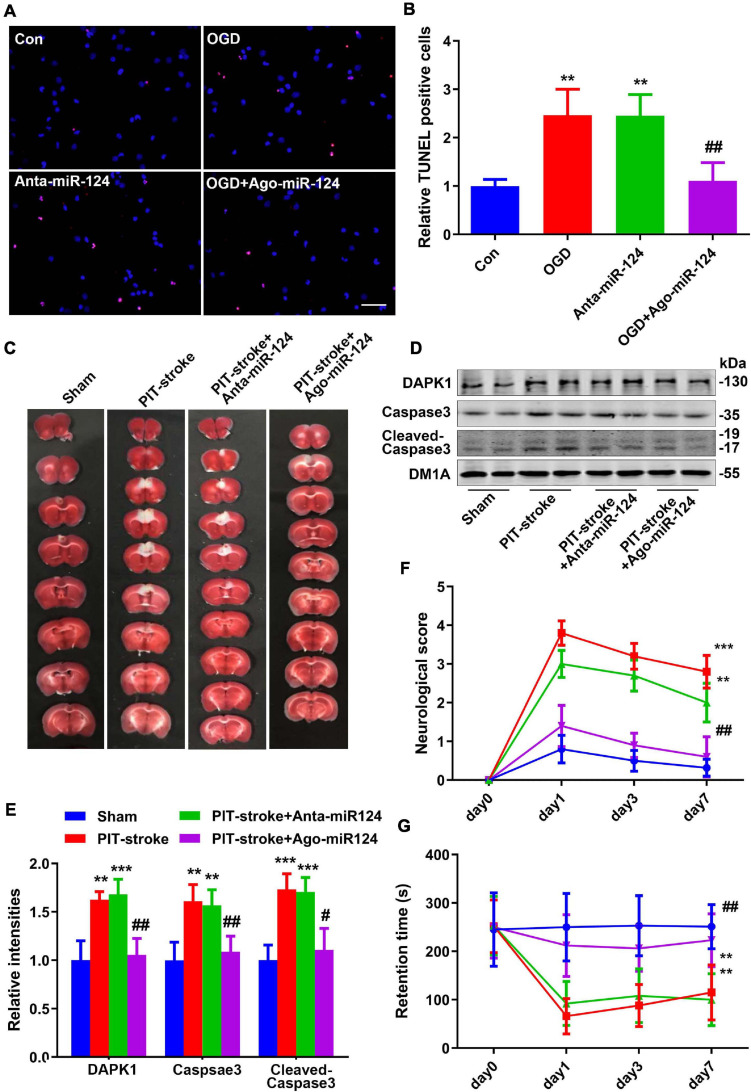
miR-124 protected against PIT-stroke damage. **(A)** TUNEL staining was performed in primary cultured neurons after OGD treatment with different miR-124 levels (scale bar = 50 μm) and quantified **(B)**. Data are presented as mean ± SD (*n* = 4/group). **p < 0.01 vs. Con, ^##^*p* < 0.01 vs. OGD group. **(C)** Representative images of TTC staining depicting Ago-miR-124-induced protection against PIT-stroke damage. Levels of DAPK1, caspase-3, and cleaved caspase-3 were measured by western blotting **(D)** and quantitatively analyzed **(E)**. Neurological scores **(F)** and performance on the rotarod test **(G)** were tested on day 1, day 3, and day 7 after PIT-stroke among different groups. Data are presented as mean ± SD (*n* = 6/group). **p < 0.01, ***p < 0.001 vs. Con, ^#^*p* < 0.05, ^##^*p* < 0.01 vs. OGD group.

To investigate the relationship between miR-124 levels and cell death, primary cultured neurons were subjected to OGD, and TUNEL staining was performed. Downregulation of miR-124 levels under normal culture conditions resulted in a similar neuron death rate compared to that for neurons subjected to OGD. Conversely, upregulation of miR-124 levels significantly rescued neuronal death following OGD (Figures 4A,B).

Next, to confirm the protective role of miR-124 in stroke damage *in vivo*, we downregulated and upregulated miR-124 levels by injecting an antagomiR (Anta-miR-124) or agomiR (Ago-miR-124), respectively, into the mouse brain. Cerebral infarction size was significantly decreased in the Ago-miR-124 + PIT-stroke group compared to that in PIT-stroke mice (Figure 4C and Supplementary Figure 3). Western blotting results revealed significant increases in DAPK1, caspase-3, and cleaved caspase-3 levels in PIT-stroke and Anta-miR-124 + PIT-stroke mice compared to that in sham mice; and significant decreases in DAPK1, caspase-3, and cleaved caspase-3 levels in Ago-miR-124 + PIT-stroke mice compared to that in PIT-stroke mice (Figures 4D,E). In addition, neurological scores and motor coordination were significantly decreased in PIT-stroke and Anta-miR-124 + PIT-stroke mice compared to that in sham mice and significantly improved in PIT-stroke + Ago-miR-124 mice compared to that in PIT-stroke mice (Figures 4F,G).

## Discussion

Cerebral ischemia induces neuronal cell death and leads to brain dysfunction (Asada et al., 2020). Preventing neuronal death is essential for protecting the brain from the detrimental effects of stroke. The mechanisms underlying cerebral ischemic cell death have been established and potential protective strategies have been identified (Graham and Liu, 2017; Ozaki et al., 2019). Nevertheless, translation of these findings to clinical practice has largely failed (Chandran et al., 2017).

After ischemia, neuronal death occurs via numerous signaling pathways, including necrosis, apoptosis, and autophagy (Liu Y. et al., 2019; Wu X. et al., 2019; Baciu et al., 2020). There have been substantial research efforts to identify the key molecules involved in neuronal death signal pathways. Previous studies have predominantly focused on elucidating changes in protein levels after cerebral ischemia (Feng et al., 2017; Lee et al., 2017). In recent years, studies have increasingly focused on the key role of non-coding RNAs in ischemia (Tiedt and Dichgans, 2018; Wolska et al., 2020). As a major component of non-coding RNAs, miRNAs in the CNS play important roles in neurogenesis, development, and maintenance of neuronal phenotype (Kaur et al., 2013; Yapijakis, 2020). Several miRNAs have been identified that play key roles in CNS disorders, including neurodegenerative and neuroimmunological disorders (Cao et al., 2016). Thus, miRNAs are considered promising targets for the treatment of CNS disorders, including cerebral ischemic stroke (Khoshnam et al., 2017).

miR-124 is one of the most abundant miRNAs in the CNS. Abnormal miR-124 levels in both peripheral blood and brain vascular endothelial cells after cerebral ischemia have been reported (Liu X. et al., 2019). Clinical and animal studies have revealed that aberrant plasma miR-124 concentrations in ischemic stroke and changes in miR-124 levels have been correlated with infarct volume (Liu X. et al., 2019). Previous studies have identified several targets of miR-124, such as PTB/hnRNP I (PTBP1) and SRY-box transcription factor (Sox9), which participate in neuronal differentiation (Faraone et al., 1993; Cheng et al., 2009); and Jagged1 (JAG1), which mediates neurogenesis in stroke (Liu et al., 2011). Moreover, cyclin-dependent kinase family 4, which is involved in motor function recovery post-stroke, has been identified as a target of miR-124 (Jeyaseelan et al., 2008). Ubiquitin-specific protease 14 (USP14), a direct downstream target of miR-124, has been shown to attenuate ischemic brain injury and promote neuronal survival in ischemic stroke (Doeppner et al., 2013; Song et al., 2019). These studies highlight the potential of miR-124 as a promising diagnostic and therapeutic target for ischemic stroke.

In this study, we first induced focal cerebral ischemia using the PIT-stroke model and detected miR-124 levels in ischemic tissues. miR-124 was downregulated 0.5 h after ischemia, an effect that persisted for 72 h, indicating that the downregulation of miR-124 may be involved in ischemic pathological processes. miR-124 is known to regulate apoptosis and autophagy in ischemic stroke (Sun et al., 2013; Liu K. et al., 2017). The decreased expression of miR-124 after ischemia observed here suggests that it may upregulate the expression of target proteins involved in stroke damage.

Previous studies have demonstrated that miR-124 reduces neuronal apoptosis and autophagy by down-regulating caspase-3 (Liu W. et al., 2017). Moreover, miR-124 inhibits oxidative stress by decreasing the levels of calpain 1/p25/cyclin-dependent kinase 5 (CDK5) in neurons (Jaenisch et al., 2016). In addition, miR-124 reduces apoptosis mediated by the accumulation of reactive oxygen species (ROS) by suppressing the annexin 5 (ANAX5)/ERK signaling pathway (Dong et al., 2018). Here, we identified that DAPK1 is a novel target of miR-124 *in vivo*. DAPK1 plays an important role in diverse apoptosis pathways, including tumor suppression and neuronal cell death (Singh et al., 2016). Indeed, DAPK1 regulates neuronal cell death at multiple levels (Kim et al., 2019). Overexpression and activation of DAPK1 impairs the vitality of nerve cells, and neurons that lack DAPK1 expression have a stronger resistance to apoptotic stimuli (Fujita and Yamashita, 2014). Moreover, DAPK1 mediates neuronal death caused by ischemic stroke via phosphorylation of the NR2B subunit of the NMDAR (Tu et al., 2010). Inhibition of DAPK1 activity confers neuroprotective effects in stroke model mice (Shamloo et al., 2005). Although miR-124 has various targets, the pathways involved in neuronal cell death are the most critical downstream players in stroke pathogenesis, and DAPK1 is a key protein involved in the cell death pathway. Hence, changes in the miR-124 target, DAPK1, post-stroke were investigated in this study.

Our data revealed a significant increase in DAPK1 protein levels in the stroke brain and OGD neurons, and reduced miR-124 levels were observed. Repression of DAPK1 by upregulation of miR-124 in N2a cells significantly reduced levels of proteins implicated in cell death, including caspase-3, cleaved caspase-3, p35/25, ERK1/2, and p-ERK1/2. Recent studies have demonstrated that these proteins participate in stroke-induced cell death, and blocking these cascades effectively reduces neuronal loss in the stroke brain (Wang et al., 2017). We also observed that upregulation of miR-124 by ago-miR-124 protected neurons against apoptotic cell death and improved behavioral performance by decreasing DAPK1 and apoptosis-related proteins, including caspase-3 and cleaved caspase-3. A previous report by Yan et al. (2020) demonstrated that miR-124 expression was significantly increased after OGD (Yan et al., 2020). This differs from our findings, possibly due to the use of primary cultured neurons vs. the N2a cells used by Yan et al. (2020). Although the specific reason for the difference is unclear, our results were confirmed by the PIT-stroke model.

Overall, our study demonstrated the key role of the miR-124/DAPK1 pathway in neuronal survival in stroke. An increase in miR-124 via downregulation of DAPK1 rescued stroke damage in mice. Our findings highlight a novel therapeutic target for ischemic stroke.

## Data Availability Statement

The raw data supporting the conclusions of this article will be made available by the authors, without undue reservation.

## Ethics Statement

The animal study was reviewed and approved by the Ethics Committee of Hunan Normal University.

## Author Contributions

YS and TT performed and analyzed the experiments and drafted the manuscript. CY and E-LC supervised the project. XY conceptualized and designed the study. All authors contributed to the article and approved the submitted version.

## Conflict of Interest

The authors declare that the research was conducted in the absence of any commercial or financial relationships that could be construed as a potential conflict of interest.
