# Corrigendum: miR-124 Alleviates Ischemic Stroke-Induced Neuronal Death by Targeting DAPK1 in Mice

**DOI:** 10.3389/fnins.2021.745087

**Published:** 2021-08-24

**Authors:** Yan Shi, Tian Tian, Er-Li Cai, Can Yang, Xin Yang

**Affiliations:** ^1^Faculty of Laboratory Medicine, School of Medicine, Hunan Normal University, Changsha, China; ^2^The Brain Cognition and Brain Disease Institute, Shenzhen Institutes of Advanced Technology, Chinese Academy of Sciences, Shenzhen, China; ^3^Shenzhen-Hong Kong Institute of Brain Science-Shenzhen Fundamental Research Institutions, Guangdong Key Lab of Brain Connectomics, Shenzhen, China; ^4^Britton Chance Center for Biomedical Photonics, Wuhan National Laboratory for Optoelectronics, Huazhong University of Science and Technology, Wuhan, China; ^5^Department of Emergency Surgery, Hubei Provincial Hospital of Integrated Chinese and Western Medicine, Wuhan, China

**Keywords:** stroke, cell death, miR-124, DAPK1, neuron

In the original article, there was a typographical error. “miR-214” should be corrected to “miR-124.”

A correction has been made to the title:

“miR-124 Alleviates Ischemic Stroke-Induced Neuronal Death by Targeting DAPK1 in Mice”

A correction has been made to the running title:

“miR-124 Alleviates Neuronal Death”

The authors apologize for this error and state that this does not change the scientific conclusions of the article in any way. The original article has been updated.

## Publisher's Note

All claims expressed in this article are solely those of the authors and do not necessarily represent those of their affiliated organizations, or those of the publisher, the editors and the reviewers. Any product that may be evaluated in this article, or claim that may be made by its manufacturer, is not guaranteed or endorsed by the publisher.

