# Correction: miR-124 alleviates ischemic stroke-induced neuronal death by targeting DAPK1 in mice

**DOI:** 10.3389/fnins.2025.1657393

**Published:** 2025-11-17

**Authors:** Yan Shi, Tian Tian, Er-Li Cai, Can Yang, Xin Yang

**Affiliations:** 1 Faculty of Laboratory Medicine, School of Medicine, Hunan Normal University, Changsha, China; 2 The Brain Cognition and Brain Disease Institute, Shenzhen Institutes of Advanced Technology, Chinese Academy of Sciences, Shenzhen, China; 3 Shenzhen-Hong Kong Institute of Brain Science-Shenzhen Fundamental Research Institutions, Guangdong Key Lab of Brain Connectomics, Shenzhen, China; 4 Britton Chance Center for Biomedical Photonics, Wuhan National Laboratory for Optoelectronics, Huazhong University of Science and Technology, Wuhan, China; 5 Department of Emergency Surgery, Hubei Provincial Hospital of Integrated Chinese and Western Medicine, Wuhan, China

**Keywords:** stroke, cell death, miR-124, DAPK1, neuron

There was a mistake in Figure 2 as published. Incorrect images were used in Figure 2A. The corrected Figure 2 appears below.

**Figure 2:**
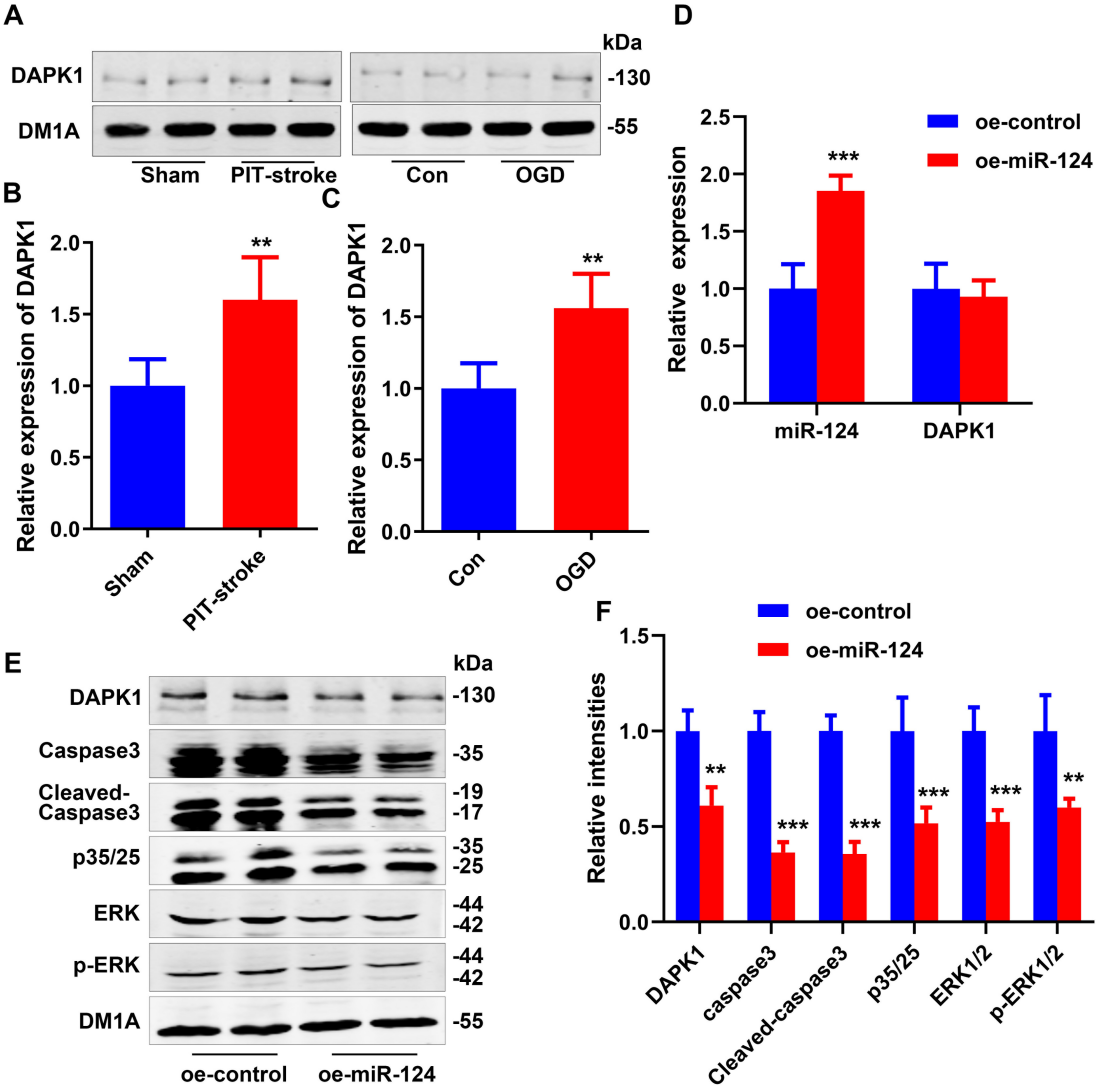
DAPK1 was identified as a target gene of miR-124. DAPK1 levels were measured by western blotting **(A)** and quantitatively analyzed **(B, C)**. Data are presented as mean ± SD (*n* = 4/group). ^**^p < 0.01, ^***^p < 0.001 vs. Con or Sham group. N2a cells were transfected with miR-124 (oe-miR-124) or control-treated (oe-control) cells. Levels of miR-124 and DAPK1 mRNA were measured by qRT-PCR **(D)**. Levels of DAPK1, caspase-3, cleaved caspase-3, p35/25, ERK1/2, and p-ERK1/2 were measured by western blotting **(E)** and quantitatively analyzed **(F)**. Data are presented as mean ± SD (*n* = 4/group). ^**^p < 0.01, ^***^p < 0.001 vs. oe-control group.

There was a mistake in Figure 4 as published. Incorrect images were used in Figure 4A. The corrected Figure 4 appears below.

**Figure 4:**
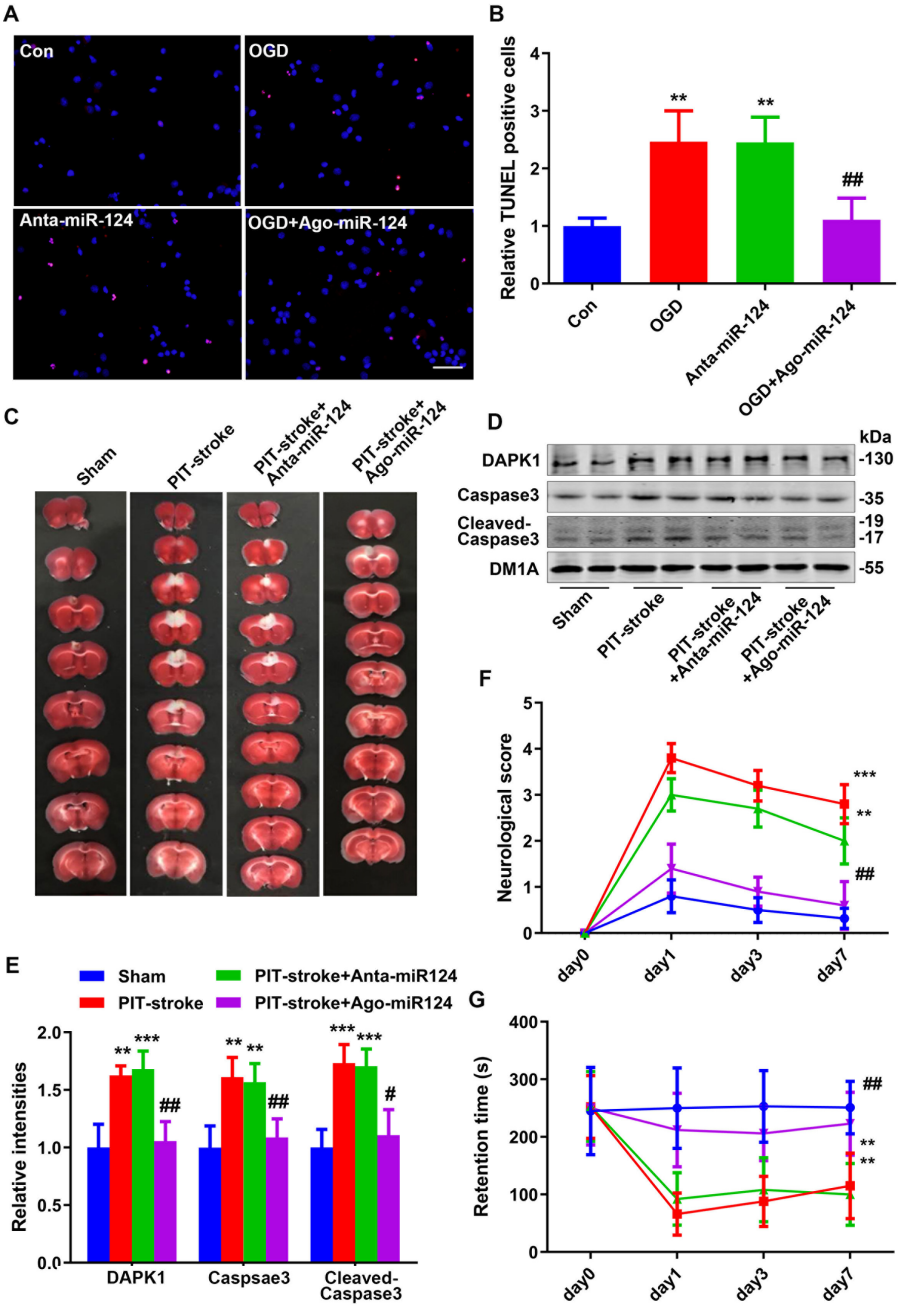
miR-124 protected against PIT-stroke damage. **(A)** TUNEL staining was performed in primary cultured neurons after OGD treatment with different miR-124 levels (scale bar = 50 μm) and quantified **(B)**. Data are presented as mean ± SD (*n* = 4/group). ^**^p < 0.01 vs. Con, ^##^*p* < 0.01 vs. OGD group. **(C)** Representative images of TTC staining depicting Ago-miR-124-induced protection against PIT-stroke damage. Levels of DAPK1, caspase-3, and cleaved caspase-3 were measured by western blotting **(D)** and quantitatively analyzed **(E)**. Neurological scores **(F)** and performance on the rotarod test **(G)** were tested on day 1, day 3, and day 7 after PIT-stroke among different groups. Data are presented as mean ± SD (*n* = 6/group). ^**^p < 0.01, ^***^p < 0.001 vs. Sham, ^#^*p* < 0.05, ^##^*p* < 0.01 vs. PIT-stroke group.

The original version of this article has been updated.

