# Supplementary material for: miR-124 Alleviates Ischemic Stroke-Induced Neuronal Death by Targeting DAPK1 in Mice
Source: Front Neurosci. 2021 Mar 26;15:649982. doi: 10.3389/fnins.2021.649982 (PMC8032895; doi:10.3389/fnins.2021.649982)

**Supplemental Figure S1**

A stable cerebral ischemia model was performed (n=3/group). The representative picture in Figure 1 is marked with a red box, and the representative picture in Figure 4 for sham group is marked with a black box.


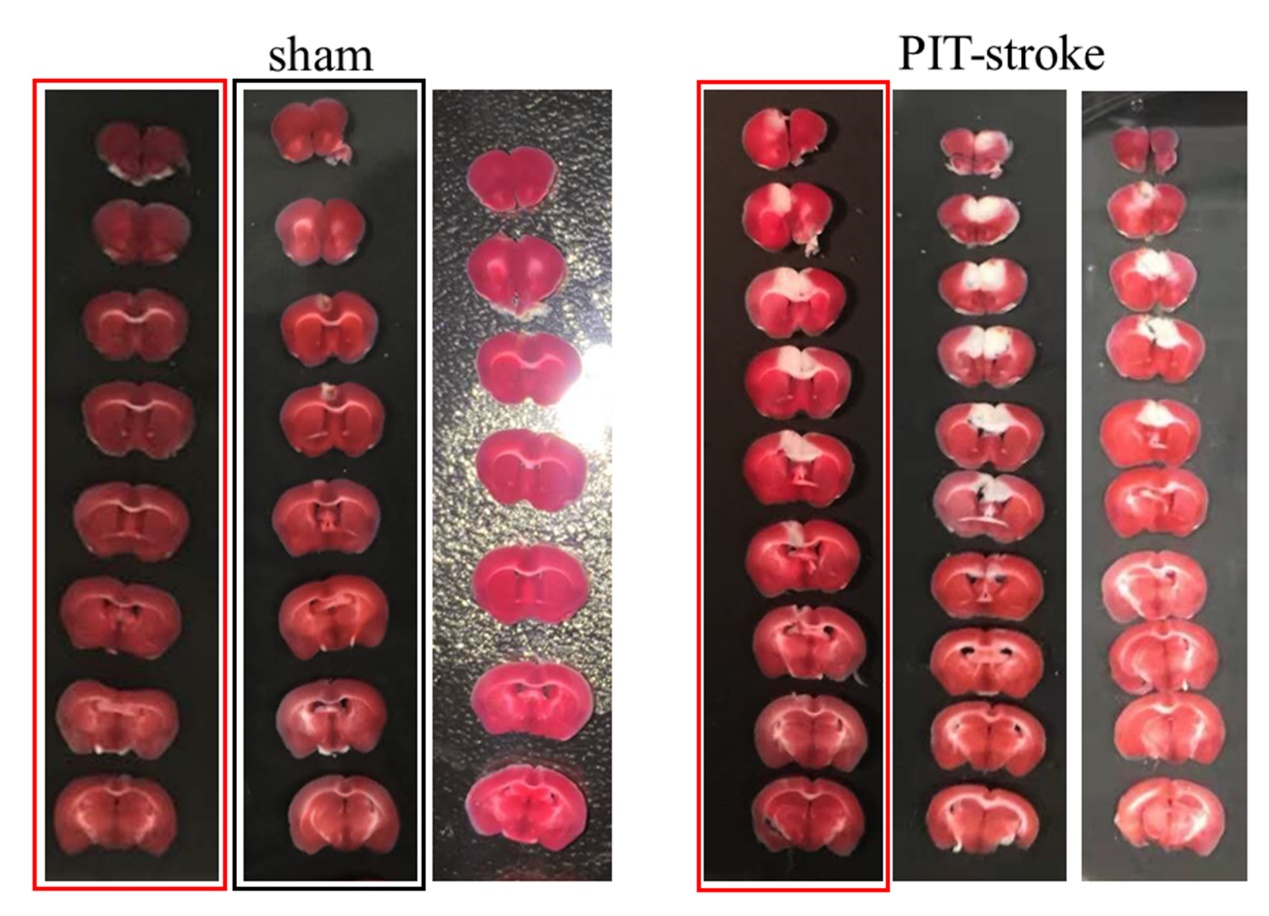


**Supplemental Figure S2**

Images of neurons were obtained using a microscope after immunofluorescence staining with an antibody recognizing microtubule-associated protein 2 (MAP2) and DAPI (Scale bar=50 μm). The proportion of MAP2-positive cells was approximately 75%.


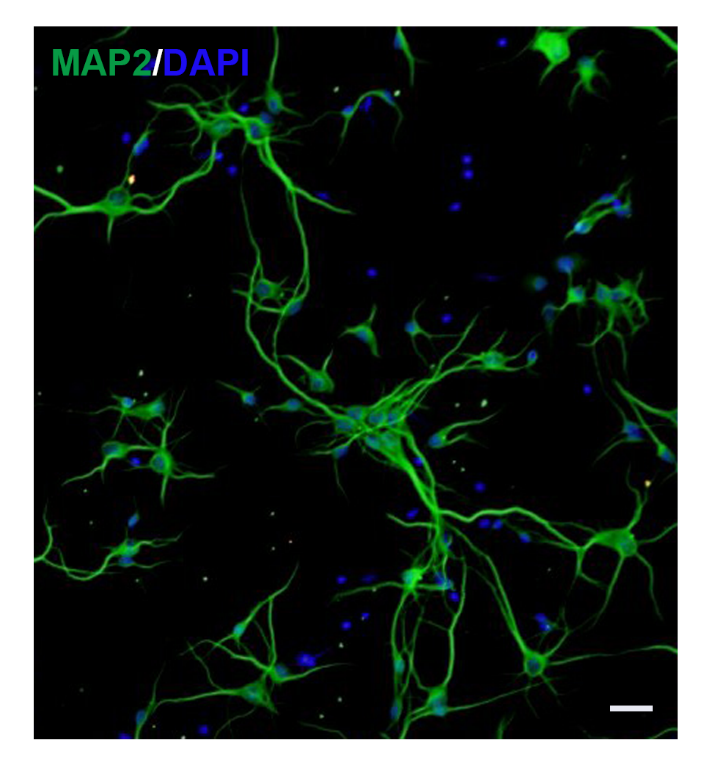


**Supplemental Figure S3**

TTC staining depicting Ago-miR-124-induced protection against PIT-stroke damage (n=4 for PIT-stroke group, n=6 for PIT-stroke+Anta-miR-124 and PIT-stroke+Ago-miR-124 group). The representative picture in Figure 4 is marked with a red box.


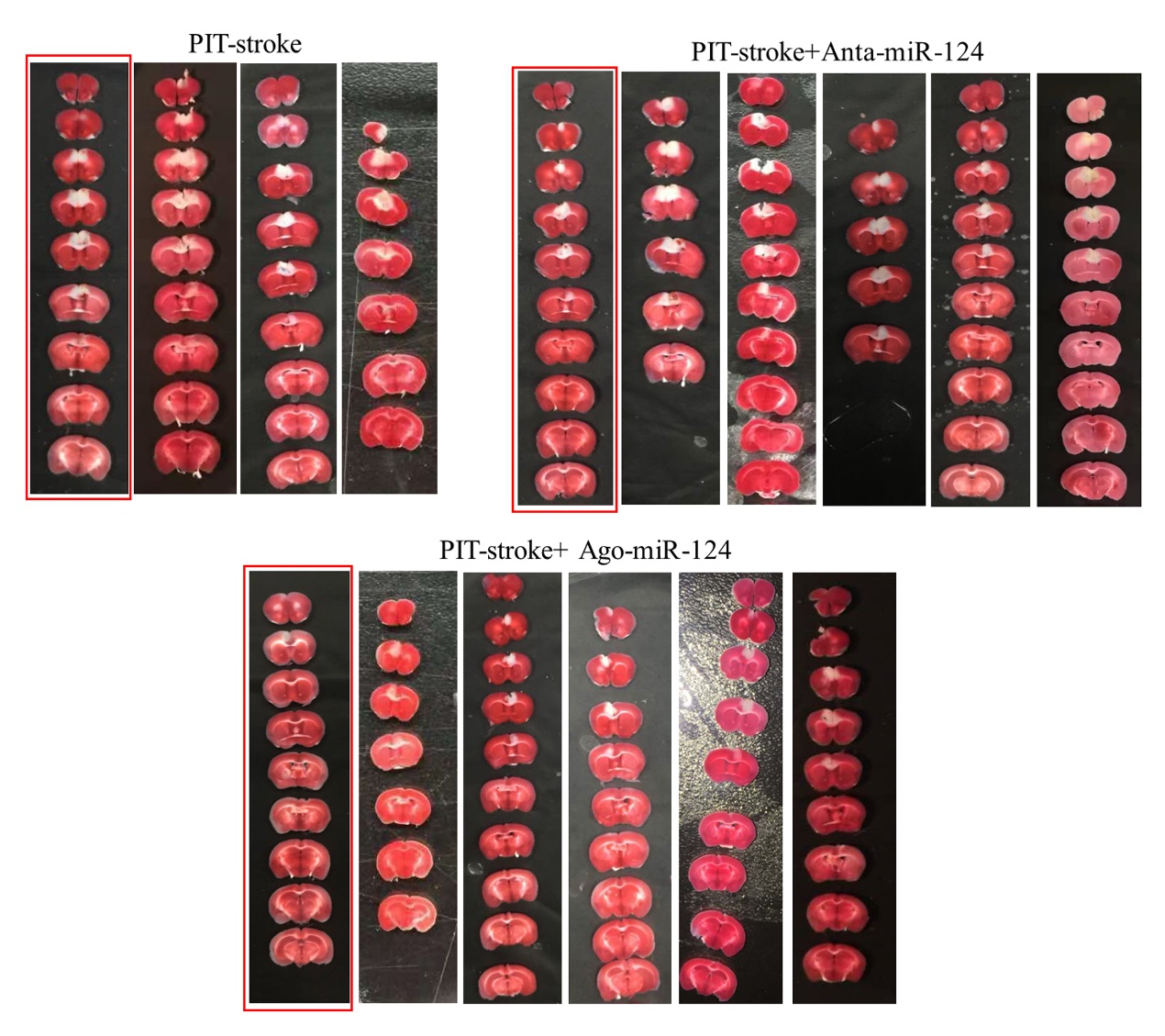

Supplement: Supplementary file 1 [file Data_Sheet_1.docx]
